# Thermal Stability, Smoke Density, and Flame Retardance of Ecotype Bio-Based Flame Retardant Agricultural Waste Bagasse/Epoxy Composites

**DOI:** 10.3390/polym13172977

**Published:** 2021-09-02

**Authors:** Shang-Hao Liu, Cing-Yu Ke, Chin-Lung Chiang

**Affiliations:** 1 Department of Ammunition Engineering and Explosion Technology, Anhui University of Science and Technology, Huainan 232001, China; u9414042@cmu.edu.tw; 2 Green Flame Retardant Material Research Laboratory, Department of Safety, Health and Environmental Engineering, Hung-Kuang University, Taichung 433, Taiwan; n74731@gmail.com

**Keywords:** agricultural waste, bagasse, flame retardant, interpenetrating network (IPN), biochar

## Abstract

In the study, agricultural waste bagasse was used as a bio-based flame retardant for reducing the flammability of epoxy. Specifically, an interpenetrating network (IPN) was formed through a ring opening reaction between the hydroxyl functional group of bagasse and the epoxy group of triglycidyl isocyanurate (TGIC), forming Bagasse@TGIC. Next, 9, 10-dihydro-9-oxa-10-phosphaphenanthrene 10-oxide (DOPO) was mixed with Bagasse@TGIC, inducing a reaction between the active hydrogen of DOPO and the epoxy group of TGIC, ultimately forming Bagasse@TGIC@DOPO with an IPN structure. Finally, the novel flame retardant was added to epoxy to create a composite. The integral procedural decomposition temperature (IPDT) of pure epoxy is 619 °C; after the introduction of the 30 wt% flame retardant, the IPDT of the resultant composite material increased to 799 °C, greatly increasing the thermal stability by 29%. After the addition of the Bagasse@TGIC@DOPO flame retardant, the limiting oxygen index increased from 21% for the pure epoxy to 29% for the composite, and the UL-94 rating improved from failing rating for the pure epoxy and V-0 rating for the composite. The Raman spectrum indicated that the addition of Bagasse@TGIC@DOPO IPN substantially increased the biochar yield during the burning process, increasing thermal stability. These results confirmed that the epoxy/Bagasse@TGIC@DOPO composite had substantial flame retarding effects.

## 1. Introduction

Epoxy (EP) is a common thermosetting material that is critical for various industries. This resin has excellent adhesiveness and chemical stability, is nonconductive, and has a low shrinkage rate. Additionally, it is commonly used in coating materials, paint, adhesives, insulation materials, aeronautical equipment, electrical equipment, and environmental applications [1,2]. However, epoxy is flammable and its combustion produces substantial poisonous gases. Therefore, fire risks for epoxy are high, limiting the use of epoxy in industries where fire is a major concern such as the aeronautical, electrical, and electronics industries. In order to improve the flame retardancy of polymer materials, traditionally, halogen flame retardants are added to make the materials less combustible. The flame retardant effect of halogen flame retardants is very good. The flame retardant mechanism is to stop the chain reaction, and the amount of addition is not too much. Flame retardants can achieve a good fire prevention effect. However, when halogens are released into the atmosphere after burning, they will destroy the ozone layer, and the amount of ultraviolet rays will increase, which is harmful to animals and plants. Additionally, the potential health and environmental hazards of halogen flame retardants have resulted in the restriction of their use. Therefore, the development of an ecologically friendly, halogen free flame retardant for epoxy is paramount [3].

Halogen flame retardants have been used extensively in flammable polymer materials. However, some of these flame retardants (e.g., PBDEs and PBB) release substantial quantities of poisonous and corrosive gases during burning. It will kill many people in the fire [4,5]. Furthermore, these poisonous and corrosive gases persist in the environment, resulting in pollution. Therefore, the addition of these hazardous substances to polymer materials has been banned. Halogen-free flame retardants are essential for life safety and environmental protection.

Most of the industrial resources are related products from petroleum, but petroleum will eventually be exhausted. We must look for resources other than petroleum. Bio-based resources are worth developing. Bio-based flame retardants, in particular agricultural waste such as rice straw [6] and rice husks [7,8] have been proposed as alternatives to the halogen flame retardant. Sugarcane bagasse is an easily obtainable agricultural waste in Taiwan and commonly used as a fertilizer which is of low value. The addition of modified sugarcane bagasse can increase a material’s mechanical properties, decrease its flammability, and reduce the required amount of other chemical flame retardants. Recycling and reuse can increase the value of agricultural waste and achieve the effect of turning stones into gold. This is in line with the concept of circular economy. Therefore, organic–inorganic composite materials are not only academically interesting, they are also useful for applications such as coatings or composite materials [9,10].

The interpenetrating polymer network (IPN) is one kind of polymer alloys consisting of two or more cross-linked polymer networks interpenetrated without or with covalent bonding. The cross-linked network structure can make the composite material more compact, so its thermal stability and flame resistance will be improved. Hu et al. showed that flame retardants were extremely efficient and greatly elevated the flame retardance of EPs, since the 0.5 wt% content could make modified EP samples pass the UL-94 V-0 rate, and with 20 wt% content the LOI value reached 49.5%, and the peak heat release rate reduced for 71.6% compared with pure EP [11].

The main purpose of this research is to use agricultural waste to develop halogen-free environmentally friendly flame retardants. In this study, sugarcane bagasse was used as a template to prepare a bio-based reactive flame retardant. Subsequently, we have mixed the resultant flame retardant with epoxy to prepare a composite material to improve the thermal properties and flame retardance of the polymer matrix. This method could be applied for safer epoxies in the transportation industry, for coating materials, creating composite materials, and creating construction materials, enabling diverse applications of polymer materials.

## 2. Materials and Methods

### 2.1. Materials

DGEBA type epoxy was kindly supplied Nan-Ya Plastics Corporation, Taipei City, Taiwan. The 4,4′-diaminodiphenylmethane (DDM) as a curing agent for epoxy and 9, 10-dihydro-9-oxa-10-phosphaphenanthrene-10-oxide (DOPO) were purchased from Sigma-Aldrich Co. Ltd., Irvine, UK. Bagasse was obtained from a local market. Isocyanurate (TGIC) was purchased from TCI, Tokyo, Japan. Anhydrous stabilized tetrahydrofuran (THF) was obtained from Lancaster Co., Morecambe, Lancashire, UK.

### 2.2. Preparation of Epoxy/Bagasse@TGIC@DOPO IPN

**Scheme 1:**
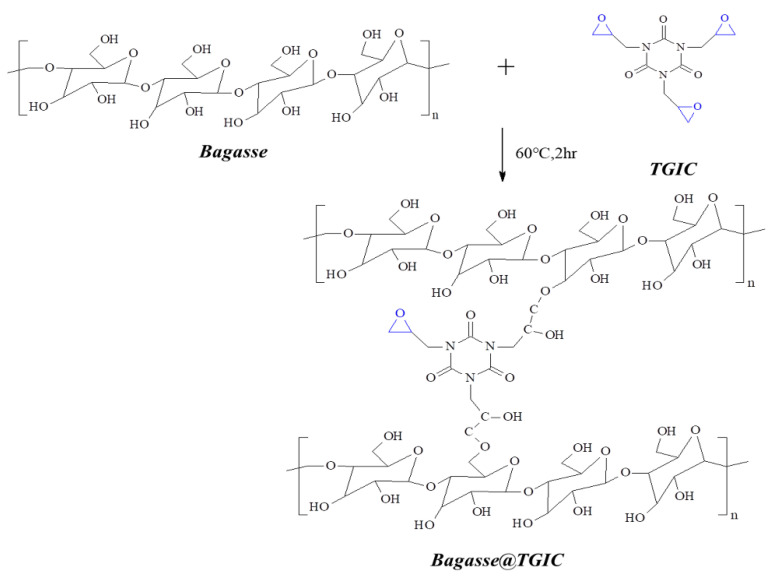
Reaction process of Bagasse@TGIC.

**Scheme 2:**
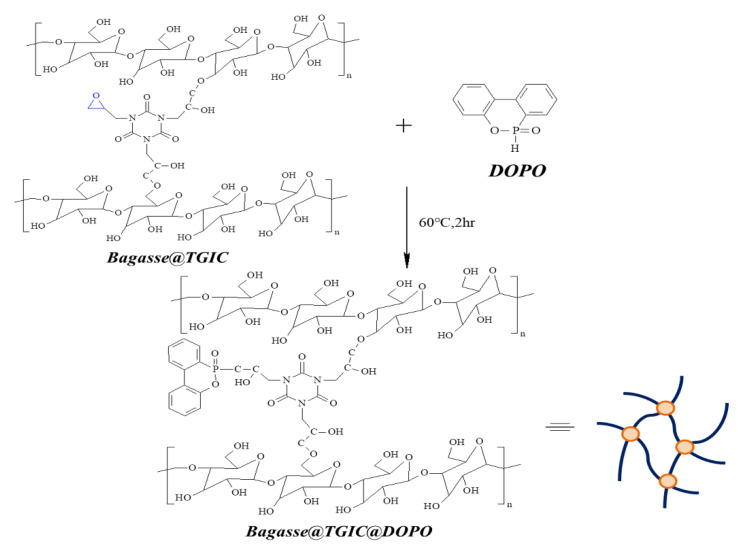
Reaction process of Bagasse@TGIC@DOPO.

**Scheme 3:**
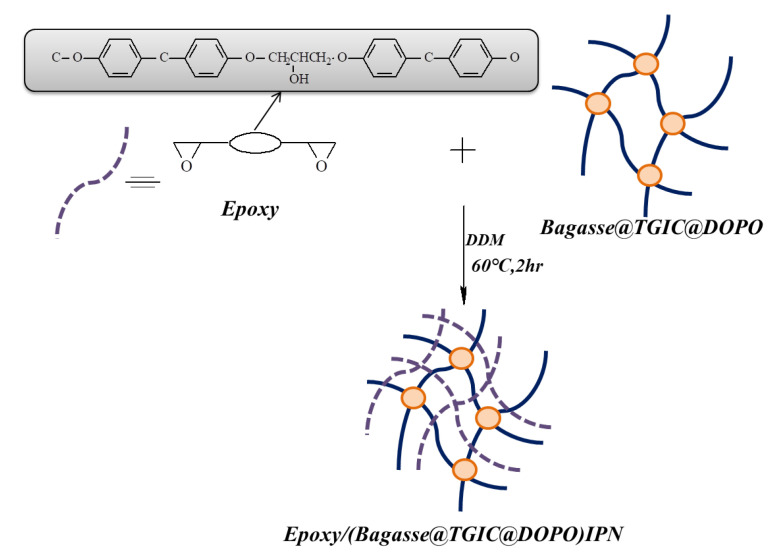
Reaction process of epoxy/Bagasse@TGIC@DOPO IPN.

First, we pulverized the bagasse, washed it with deionized water at 100 °C three times, filtered it three times, and put it in an oven to remove the water at 100 °C. We put bagasse (1.366 g) and triglycidyl isocyanurate (TGIC; 1.67 g) into a 100 mL serum bottle and added 80 mL of tetrahydrofuran solvent for modification and reaction at 60 °C for 2 h. This is solution A, as presented in Scheme 1. We put DOPO (2.428 g) into solution A and reacted this at 60 °C for 2 h to obtain a Bagasse@TGIC@DOPO flame retardant, which is solution B, as displayed in Scheme 2. Solution B was poured into the epoxy matrix (10 g), stirred at 60 °C for 2 h before the hardener DDM (2.75 g) was added, and observed whether viscosity increased to 10,000 cP, after which it was poured into a mold, placed at room temperature for 24 h, and placed in an oven at 60 °C. The temperature increased from 20 to 180 °C to form the composite materials, as illustrated in Scheme 3. 

### 2.3. Measurements

The thermal degradation of composite was examined using a thermogravimetric analyzer (TGA) (Perkin Elmer TGA 7) from room temperature to 800 °C at a rate of 10 °C/min under an atmosphere of nitrogen. The measurements were made on 6–10 mg samples. Weight-loss/temperature curves were plotted. 

A smoke density test was conducted in an FTT 0064 NBS smoke density test chamber (Fire Testing Technology Ltd., West Sussex, UK) according to ISO 5659-2. The size of the chamber is 914 × 914 × 610 mm. The sample (65 × 65 mm) was exposed to a radiant heat of 50 kW/m^2^ in flameless combustion mode at a temperature of 560 °C for 1200 s. 

The LOI is defined as the minimum fraction of O_2_ in a mixture of O_2_ and N_2_ that will just support flaming combustion. The LOI test was performed according to the testing procedure of the ASTM D 2836 Oxygen Index Method, with a test specimen bar 7–15 cm long, 6.5 ± 0.5 mm wide, and 3.0 ± 0.5 mm thick. The sample bars were suspended vertically and ignited by a Bunsen burner. The flame was removed and the timer was started. The concentration of oxygen was increased if the flame on the specimen was extinguished before burning for 3 min or burning away 5 cm of the bar. The oxygen content was adjusted until the limiting concentration was determined. The vertical burning test was done inside a fume hood. Samples were held vertically with tongs at one end and burned from the free end. Samples were exposed to an ignition source for 10 s then they were allowed to burn above a cotton wool until both the sample and cotton wool extinguished. Observable parameters were recorded to assess fire retardance. The UL 94 test classifies the materials as V-0, V-1, and V-2 according to the time period needed before self-extinction and the occurrence of flaming dripping after removing the ignition source. V-0 is the most ambitious and desired classification. 

The morphology of the fractured surface of the composites was studied under a scanning electron microscope (SEM) (JEOL JSM 840A, Japan). Raman spectra were recorded using a Lab Ram I confocal Raman spectrometer (Dilor, France). A He–Ne laser with a laser power of about 15 mW at the sample surface was utilized to provide an excitation wavelength of 632.8 nm. A holographic notch filter reflected the exciting line into an Olympus BX40 microscope, Tokyo, Japan.

## 3. Results and Discussion

### 3.1. Thermal Properties

**Figure 1:**
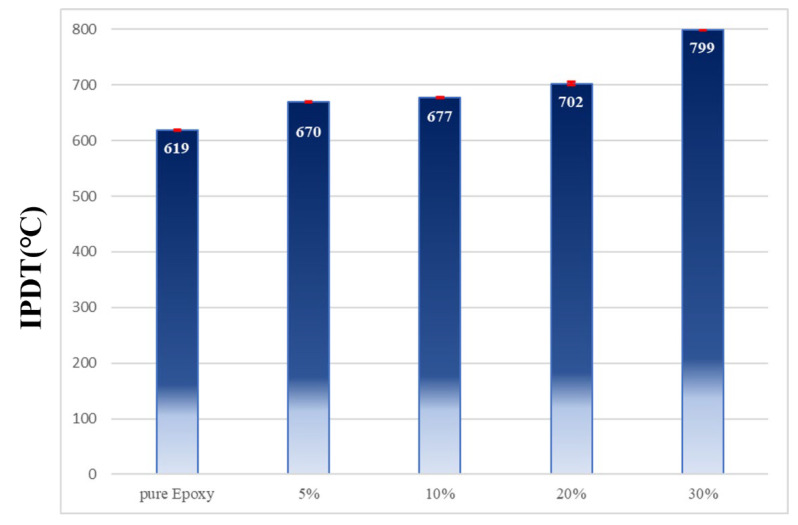
IPDT of pure Epoxy and Epoxy/Bagasse@TGIC@DOPO IPN composites.

The integral procedural decomposition temperature (IPDT) [12] is a common indicator used for evaluating the thermal stability of materials. Two factors influence the IPDT: Initial pyrolysis temperature and the biochar yield. High IPDT indicates high thermal stability. Figure 1 compares the IPDT of tested materials. The IPDT of pure epoxy was determined to be 619 °C. After the addition of Bagasse@TGIC@DOPO at 30 wt%, the IPDT of the composite materials increased to 799 °C, an increase of 180 °C compared to the pure epoxy. Thus, the addition of Bagasse@TGIC@DOPO enhanced the thermal stability of the epoxy composite materials. The behavior is due to the fact that the biochar layer produced after the pyrolysis protected the polymer matrix and increased the thermal stability of the composites. 

### 3.2. Kinetics of Thermal Degradation

Polymer materials can burn after absorbing thermal energy, producing gases and coke. However, an energy barrier (i.e., the activation energy) must be overcome before pyrolysis begins. The pyrolysis kinetics of Epoxy/Bagasse@TGIC@DOPO IPN composite materials under a nitrogen environment were computed, and the changes in activation energy were analyzed by testing at different heating rates of 5, 10, 20, and 40 °C/min.

**Figure 2:**
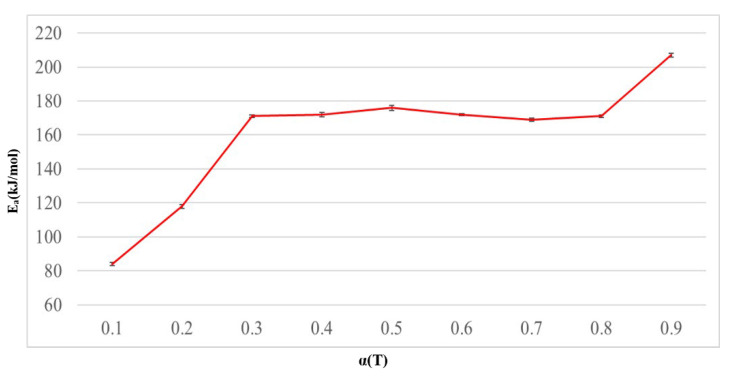
Activation energies (Ea) as a function of conversion for Epoxy/Bagasse @TGIC@ DOPO IPN 30% determined through Ozawa’s method.

The results obtained using Ozawa’s method [13] are compiled and presented in Table 1. The results revealed that most of the R values for a linear regression of the Epoxy/Bagasse@TGIC@DOPO IPN 30% composite material exceeded 0.99, indicating that this method is suitable for the material in this study. Data in Figure 2 and Table 1 indicated that the structure of Bagasse@TGIC@DOPO contained phosphorus, whereas Bagasse contained elements such as lignin, cellulose, and hemicellulose. In general, phosphorus has the lowest pyrolysis temperature of 300 °C and forms char [14]. Next, hemicellulose undergoes pyrolysis 210–370 °C, and cellulose would undergo pyrolysis between 260–410 °C. Lignin contains various aromatic rings, these rings form a complex aromatic ring network that inhibits pyrolysis. Therefore, lignin pyrolyzes at 600 °C [15]. Thus, the activation energy of Epoxy/Bagasse@TGIC@DOPO IPN 30% composite materials increase as the conversion rate increases, this phenomenon indicates that the produced biochar protected the polymer matrix.

### 3.3. Smoke Density Test

To further explore the influence of Bagasse@TGIC@DOPO flame retardant for suppressing smoke from pure epoxy, smoke density tests were conducted for the neat epoxy and Epoxy/Bagasse@TGIC@DOPO IPN 30% composite material. Data were collected regarding the smoke production rate [16,17].

The smoke density (Ds) is defined as follows [18]:

$$\mathrm{Ds}=\frac{V}{A\times L}\log\frac{100}{T}$$

V is the smoke box volume (m^3^);
A is the sample exposure area (m^2^);
L is the optical path length (m);
T is the transmittance rate (%).

**Figure 3:**
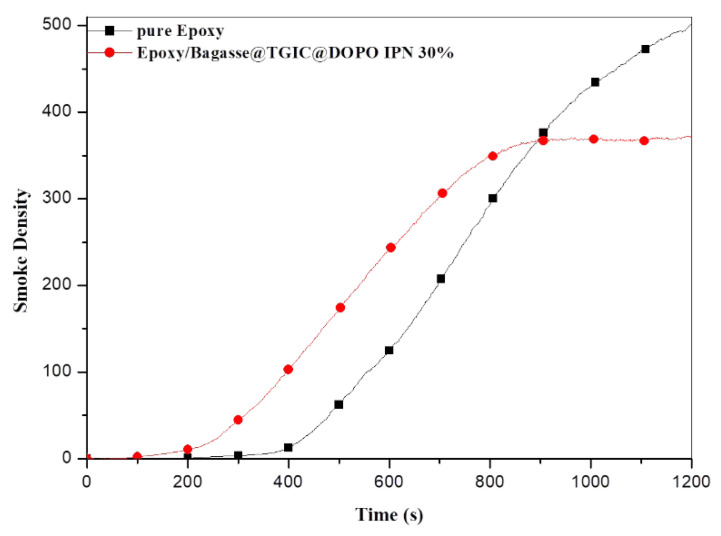
Smoke density curves of pure epoxy and Epoxy/Bagasse@TGIC@DOPO IPN 30% composites.

Figure 3 reveals that the smoke density of pure epoxy increased from 163 to 1200 s, and a reduction in rate was not observed. The greatest smoke density of the pure epoxy was 501.1 at 1200 s. An observation of the Epoxy/Bagasse@TGIC@DOPO IPN 30% composite material revealed that the initial appearance of smoke for the composite material was 80 s earlier than that of pure epoxy, and a reduction in smoke production rate was observed at 800 s. The maximum smoke density of the composite material was 373.3 at 1200 s and lower than that of pure epoxy at the same time. The low smoke density increases safety during fire due to the higher visibility [19]. The phosphorus in Bagasse@TGIC@DOPO flame retardant pyrolyzed at a lower temperature, releasing noncombustible gas and diluting oxygen in the air, resulted in the 80 s slower smoke onset. In the condensed phase, the phosphorus in the structure produced phosphoric acid and polyphosphoric acid, which underwent an epoxy reaction that involved esterification, dehydration, and decomposition. Accordingly, a char layer with polycyclic aromatic structure bridged by P-O-C and P-O was formed. The char layer effectively prevented the transfer of heat, oxygen, combustible gas, and smoke particles and thus prevented the combustion of pure epoxy [20,21]. This mechanism may explain the substantial smoke suppression effect of the Bagasse@TGIC@DOPO flame retardant on the pure epoxy.

### 3.4. Flame Retardance

The UL-94 rating measurement involves igniting a material multiple times and measuring the total burn time using a flame burning standard test strip. The flame retardance of the material is determined by placing a cotton below the sample and observing whether flaming particles from the material ignite the cotton, results are classified as V-0, V-1, and V-2. V-0 is the highest rating. Measurement of the limiting oxygen index (LOI) involves determining the flame retardant level of a high polymer material by observing differences in oxygen and nitrogen concentrations. The atmospheric content of oxygen is approximately 21%. Materials with LOI ≥ 21 burn freely in the air, materials with LOI 22–25 extinguish immediately when burned, materials with LOI ≥ 26 are difficult to be ignited [22]. The flow velocity of oxygen was in mL/s. The formula for LOI was as follows:

$$\mathrm{LOI}=\frac{O_{2}}{O_{2}+N_{2}}\times 100$$

**Figure 4:**
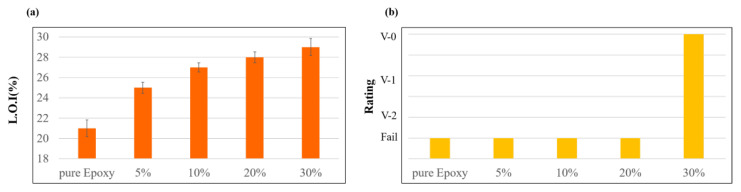
Effect of various Bagasse@ TGIC@DOPO contents on the (**a**) LOI and (**b**) UL-94 of Epoxy/Bagasse@TGIC@ DOPO IPN composites.

Figure 4a reveals that the LOI of pure epoxy is 21%, making it an easily combustible polymer material. Tests with oxygen concentrations revealed that the Epoxy/Bagasse@TGIC@DOPO IPN 30 wt% composite material had an LOI of 29%, substantially increasing flame retardance. Additionally, Figure 4b showed that pure epoxy burned and failed the UL-94 rating tests. At flame retardant levels of 30 wt%, the Epoxy/Bagasse@TGIC@DOPO IPN composite material extinguished within 10 s, achieving a V-0 rating. This result also indicates that the Bagasse@TGIC@DOPO flame retardant has high performance. The mechanism for this property is due to phosphorus in the structure vaporizing during pyrolysis and capturing free radicals, as well as promoting the formation of char during the condensed phase [23]. Nitrogen in the structure releases noncombustible gases during pyrolysis, causing the inflation of the char layer, and preventing the flame from spreading [24]. The benzene ring of the bagasse containing lignin facilitates char generation, and the char layer could effectively prevent high-temperature burning.

### 3.5. Morphology

**Figure 5:**
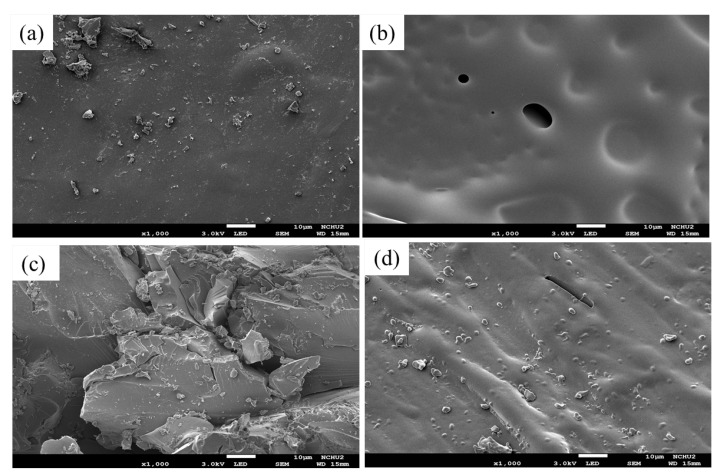
SEM micrographs of composites (**a**) Epoxy/Bagasse@ TGIC@DOPO IPN 5% (before burning) (×1K); (**b**) Epoxy/Bagasse@TGIC@DOPO IPN 5% (after burning) (×1K); (**c**) Epoxy/Bagasse@TGIC@DOPO IPN 30% (before burning) (×1K); (**d**) Epoxy /Bagasse@TGIC@DOPO IPN 30% (after burning) (×1K).

The morphology was obtained using an analytical field emission scanning electron microscope from Figure 5. The surface microtexture, grain size, and surface conditions of the composite materials were observed. Figure 5a indicated that Epoxy/Bagasse@TGIC@DOPO IPN 5% commences before burning, some flame retardant particles appeared on the material surface due to the trace amounts of additive. Figure 5b reveals that biochar layers begin to form after surface burning of Epoxy/Bagasse@TGIC@DOPO IPN 5%. However, the small amount of biochar formed led to a poor retarding effect, the UL-94 results were correspondingly poor, and the material failed the test. Figure 5c reveals that before burning of Epoxy/Bagasse@TGIC@DOPO IPN 30% commences, unevenness could clearly be observed on the surface due to the increased additive. Figure 5d indicates that compact and dense biochar layers began to cover the surface of Epoxy/Bagasse@TGIC@DOPO IPN 30% after the surface burning process. These layers are formed since phosphorus has dehydrated and formed char, and nitrogen groups have released noncombustible gases during the burning process, causing the expansion of the char layer and preventing it from spreading [25]. Finally, the benzene ring facilitates char generation, blocking transmission between gases and the fire source, and enhancing the thermal stability of the material.

### 3.6. Char Analysis

The Raman spectrum is a useful method for analyzing different types of carbon materials, and in particular post-burning char. Raman analysis was performed on the biochar obtained from placing Epoxy/Bagasse@TGIC@DOPO IPN composite materials in a 800 °C furnace for 1 and 5 min. The disordered band (D-band) is located at 1350 cm^−1^ and indicated a carbon structure with an irregular aliphatic fatty group with an sp^3^ structure. The graphitic band (G-band) is located at the 1580 cm^−1^ wave band, and indicates a hexa-atomic aromatic carbon ring structure with an sp^2^ structure [26]. After burning, D-band substances are converted to G-band substances, and a ratio indicating the extent of carbonization was obtained by dividing the areas (D/G).

**Figure 6:**
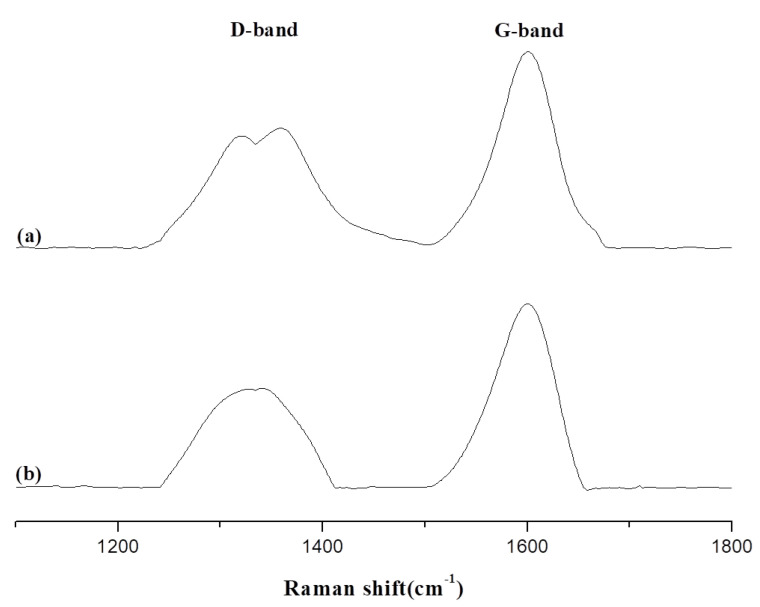
Raman spectra of biochar products from Epoxy/Bagasse@TGIC@DOPO IPN 5% at 800 °C (**a**) 1 min, (**b**) 5 min.

**Figure 7:**
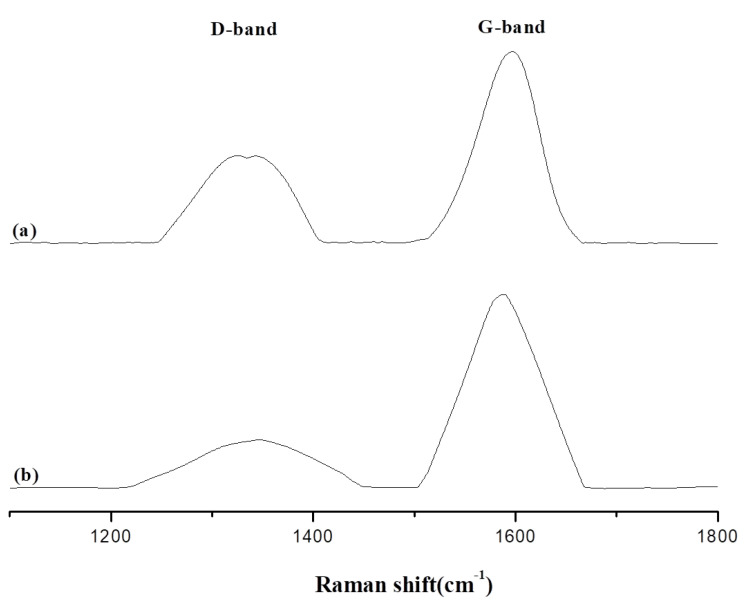
Raman spectra of biochar products from Epoxy/Bagasse@TGIC@DOPO IPN 30% at 800 °C (**a**) 1 min, (**b**) 5 min.

Changes in and analysis of D- and G-bands of Epoxy/Bagasse@TGIC@DOPO IPN 5% and Epoxy/Bagasse@TGIC@DOPO IPN 30% are presented in Figure 6 and Figure 7, and the results are compiled in Table 2. The results in Table 2 indicate that for Epoxy/Bagasse@TGIC@DOPO IPN 5%, D/G values after 1 and 5 min burning were 1.05 and 0.82, respectively. For Epoxy/Bagasse@TGIC@DOPO 30%, D/G values after 1 and 5 min were 0.63 and 0.38, respectively. Since Bagasse@TGIC@DOPO contains trace amounts of silicon, biochar was formed on the surface during the burning process [27]. Moreover, DOPO would produce phosphoric acid during pyrolysis, phosphoric acid acts as a dehydration agent and promotes biochar formation [28,29]. Therefore, substantial biochar was produced during the burning process, enhancing the thermal stability and anti-oxidative properties of the material, increasing the additive content, and resulting in a greater flame retardant effect.

## 4. Conclusions

This research has successfully used bagasse as a reaction template to successfully graft phosphorus and nitrogen-containing compounds onto its surface to form a new type of halogen-free flame retardant. In addition to decontaminating agricultural waste, it also can enhance its economic value. Through the calculation of IPDT and activation energy of thermal degradation, we can prove that the new flame retardant can significantly improve the thermal stability of epoxy resin. The experimental data can show that the new flame retardant material has the effect of suppressing smoke through the smoke concentration test. Both UL94 and LOI can show that the final composite material has excellent flame retardancy. Through the observation of SEM morphology and the in-depth discussion of RAMAN spectroscopy, we can understand the flame retardant mechanism of this flame retardant, which improves the flame retardancy of the material through the flame retardant mechanism of the condensed phase. Overall, experimental results indicated that the developed Bagasse@TGIC@DOPO IPN flame retardant in the current study has a substantial fire retarding effect.

## Figures and Tables

**Table 1:** The calculated activation energy of thermal degradation with various conversions by Ozawa’s method.

α	Epoxy/Bagasse@TGIC@DOPO IPN	
	E (kJ/mole)	R Value
0.1	843.3 ± 0.8	0.98
0.2	118.2 ± 1.1	0.98
0.3	171.2 ± 0.7	0.99
0.4	172.3 ± 1.2	0.99
0.5	176.3 ± 1.5	0.99
0.6	172.2 ± 0.4	0.99
0.7	169.3 ± 0.7	0.99
0.8	171.3 ± 0.7	0.99
0.9	207.1±1.3	0.99
△E(av)	160.3 ± 0.3	

**Table 2:** D/G for the Raman shift from Epoxy/Bagasse@TGIC@DOPO IPN composites biochar.

Sample No.		D-Band	G-Band	D/G
		1350 cm^−1^	1580 cm^−1^	
Epoxy/Bagasse@TGIC@DOPO IPN 5%	1 min	33,063	31,415	1.05
	5 min	60,288	73,553	0.82
Epoxy/Bagasse@TGIC@DOPO IPN 30%	1 min	108,296	171,453	0.63
	5 min	65,277	172,587	0.38

## Data Availability

The data presented in this study are available on request from the corresponding author.
